# Toward laser-induced tuning of plasmonic response in high aspect ratio gold nanostructures

**DOI:** 10.1515/nanoph-2022-0193

**Published:** 2022-08-08

**Authors:** Mario Pelaez-Fernandez, Bruno Majérus, Daniel Funes-Hernando, Romain Dufour, Jean-Luc Duvail, Luc Henrard, Raul Arenal

**Affiliations:** Instituto de Nanociencia y Materiales de Aragon (INMA), CSIC- U. de Zaragoza , Calle Pedro Cerbuna 12, Zaragoza 50009, Spain; Laboratorio de Microscopias Avanzadas, Universidad de Zaragoza, Calle Mariano Esquillor, Zaragoza 50018, Spain; Laboratoire de Physique du Solide, NISM, University of Namur, 61, Rue de Bruxelles, Namur 5000, Belgium; Institut des Matériaux de Nantes Jean Rouxel, CNRS-Université de Nantes, Nantes, France; ARAID Foundation, Zaragoza 50018, Spain

**Keywords:** gold nanostructures, laser, optoelectronics, plasmonics, tuning

## Abstract

High aspect-ratio gold nanostructures sustain Fabry–Perot-like surface plasmon responses from infrared to visible light energies. We show that some resonances can be tuned by means of laser irradiation, where low energy modes stay unperturbed. After laser irradiation, gold nanowires’ tips are transformed into nanoparticles of various sizes joint to gold nanowires, producing high aspect-ratio half-dumbbells and dumbbells structures. The plasmonic behaviour of both the nanowires and the newly created nanostructures has been characterised by in-depth monochromated electron energy loss spectroscopy (EELS) developed in a transmission electron microscope (TEM) and state-of-the-art discrete dipole approximation (DDA) calculations. All these analyses serve as experimental proof of the selective tuning (or robustness) of the plasmonic modes of the nanostructures in a specific spectral range, which is of critical interest regarding applications for sensing devices, nano-sources or nanophotonic waveguide, as well as optical remote control.

## 1 Introduction

Ever since the term plasmonics was coined almost two decades ago [1, 2], the research surrounding this topic has become an incredibly fertile research field, mainly for biotechnology [3], electronics, photonics [4], photovoltaic energy [5] and chemical analysis [6]. In this sense, the study and the tuning of localised surface plasmon resonances (LSPRs) of metallic nanosystems have been a cornerstone of plasmonics due to their various realms of application; ranging from photonics to electronics, sensors [7–9] or even chemical analysis through surface-enhanced Raman scattering (SERS) [10–13]. These localised charge resonances occur at the surface of these nanostructures when excited with an external electromagnetic field (such as light or electron beams).

Low-loss electron energy loss spectroscopy (EELS) is a very fitting technique for studying this kind of phenomena in these nanostructures given its nanometric spatial resolution and an energy resolution that can go down to as low as a few meV [14–19].

This extensive research has delved into the plasmonic behaviour of a myriad of nanostructures, extended but not limited to metallic nanoparticles of various shapes [14, 20], [21], [22], [23], core–shell structures [24] and, of course, higher aspect-ratio nanostructures such as nanotubes [25, 26] nanorods [17, 27], [28], [29], [30] and nanocarrots [31]. Amongst all these nanostructures, metallic nanowires have attracted special interest [32–36] given their potential use as nanophotonic waveguides, allowing for a much smaller circuitry than their glass counterparts [2, 36, 37]. The high aspect ratio in nanowires also allow to tune the plasmonic resonance to lower energies [38] or to lower the excitation damping [39]. These FP resonances for finite size NW have been shown to follow the dispersion relations close to that of an infinite NW, following the basic ideas of Fabry–Pérot (FP) interference or standing waves [35, 36, 38, 39], which has also been of significant interest [40].

Furthermore, recent studies have shown the possibility to modify the morphology of these nanowires by means of laser irradiation, providing a new opportunity to tune the response of high aspect-ratio nanostructures [41, 42]. However, the study of very high aspect ratio Au nanostructures is quite experimentally challenging because they sustain many high wavelength FP modes taking place at low energies (down to 0.1 eV) and most examples found in the literature present a much lower aspect-ratio besides very recent exceptions for Cu nanowires [43].

Our present EELS studies illustrate how a vast number of modes can be analysed and mapped with state-of-the-art data science tools [44, 45] in diverse high aspect-ratio plasmonic nanostructures (namely, plain nanowires, half-dumbbells and dumbbells), providing the opportunity to investigate not only very high aspect ratio NW but also the role of the morphology of the extremities of the NW on the FP modes. With the support of numerical simulations, we found that, whereas some of the FP modes are extremely robust against such changes of morphology, higher energy (small wavelength) modes are affected and can be tuned by a modification of the shape of their extremities, e.g., by means of irradiation. This is of great interest for nano-waveguiding and sensing nanodevices.

The large momentum (small wavelength) FP modes associated with the propagating surface plasmon polariton (SPP) of infinite NW and the LSPR of lower aspect ratio nanosystems such as the one of the nanoparticles obtained after laser irradiation have overlapping resonance energies. Based on our custom data analysis methodology, we have been able to resolve the contribution of both contribution in a very narrow energy range (2.3–2.5 eV).

From both an experimental and a modelling point of view, our present studies illustrate how the plasmonic response of high aspect ratio gold 1D nanostructures can be tuned by means nanoparticles attached to the tips of gold nanowires, which can be created by means of laser irradiation [32]. These works serve as well as a proof of concept as to how low-loss EELS can be used to understand plasmonic coupling in these particular nanostructures. These studies will have an impact on real applications of such nanostrucures as sensors [13] and nanophotonics [40].

## 2 Results and discussion

**Figure 1:**
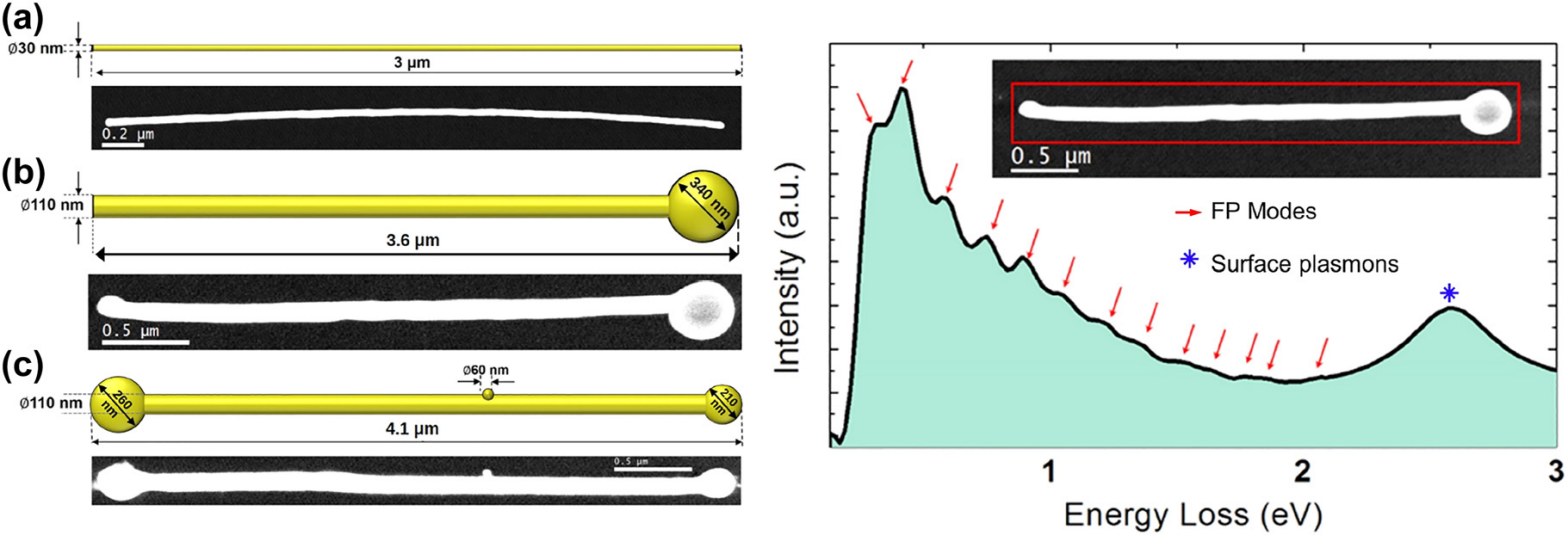
Left: Scaled comprehensive sketch (top) and STEM-HAADF micrograph (bottom) for (from top to bottom) an Au NW, an Au half-dumbbell and an Au dumbbell nanostructure. Right: Low-loss EELS zero-loss peak (ZLP) removed integrated spectra on the half-dumbbell high aspect-ratio nanostructure. Features related to FP modes and the surface plasmons are marked by arrows and by an asterisk, respectively. A STEM-HAADF micrograph featuring the area on which the spectrum has been integrated (red rectangle) is shown as an inset.

Figure 1 shows a comprehensive view of the geometry of the three nanostructures studied in the present paper, featuring a scaled sketch and a high-angle annular dark-field (HAADF) scanning transmission electron microscope (STEM) micrograph for each nanostructure. Details on the fabrication of these nanostructures can be found in the literature [13, 46] and in the Supplementary Information. The integrated low-loss EEL spectra of the half-dumbbell nanostructure after applying a custom background extraction routine (see Supplementary Information) is also displayed in Figure 1. As we can see, the integrated low-loss EEL spectra of these nanostructures show two distinct types of features. On the one hand, we can find many narrow features (with a full width half maximum (FWHM) of ∼0.2 eV) in the spectral window from 0.2 to 2 eV. These features have been assigned in the literature to the nanostructure behaving like a quantified Fabry–Pérot resonator [35, 38, 39]. On the other hand, the spectra also present one (or several) features at high energies (2.3–2.4 eV) related to other gold surface plasmon modes of either the NW and their extremities. This is a crucial part of these works since these relate to the coupling or lack thereof in the nanostructure being measured.

### 2.1 Gold nanowires with high aspect ratio

**Figure 2:**
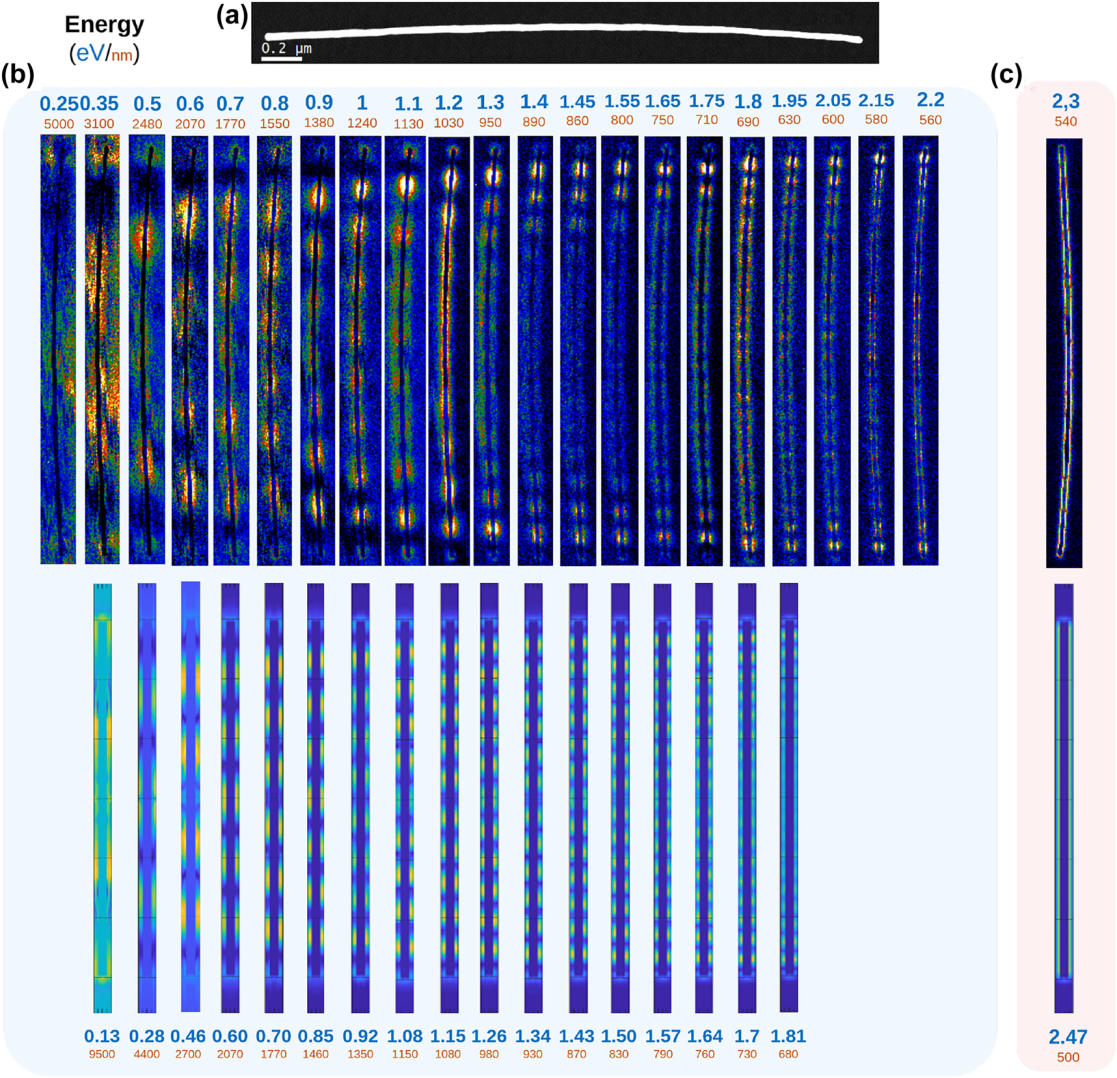
Spatial distribution of the plasmon modes of nanowire obtained by background extraction and NMF decomposition. (a) STEM-HAADF micrograph. (b) Top: NMF components corresponding to FP modes in ascending order of resonance m energy. Bottom: DDA simulations corresponding to FP modes with the same number of nodes as their experimental counterparts. (c) Top: NMF components corresponding to the surface mode of the Au NW. Bottom: Corresponding DDA simulations.

Figure 2 shows the experimental and simulated EELS maps of a high aspect-ratio Au NW with a length of 3 μm long and a diameter of 30 nm. Our non-negative matrix factorisation (NMF) decomposition of the EELS spectrum-image (SPIM) [47, 48] allows for a clear distinction between the Fabry–Pérot modes, which can be discerned from 0.3 to 2.2 eV, and a continuous surface mode at 2.3 eV. The EELS features are very well reproduced by the numerical simulations.

A wavelength and thus a wavevector *k* can be associated with each FP excitations (*k* = (*n* − 1)⋅*π*/*L*_AN_) were *n* is the number of anti-nodes (excluding the tips) of the EELS map and *L*_AN_ is the distance between the two furthest antinodes being measured (See Supplementary Information). We note that no observable wavelength contraction phenomena has been observed neither in the experimental analysis nor in the simulated data, in contradiction to what has been previously reported [17, 36, 38] with the exception of the distance between the out-most antinodes of the maps and the tips of the NW, which turned out to be shorter than the rest of the distances measured, as we will discuss later. We note that other experimental studies do not mention the observation of wavelength contraction [43] and that the modelling does not support this contraction (see Supplementary Information).

**Figure 3:**
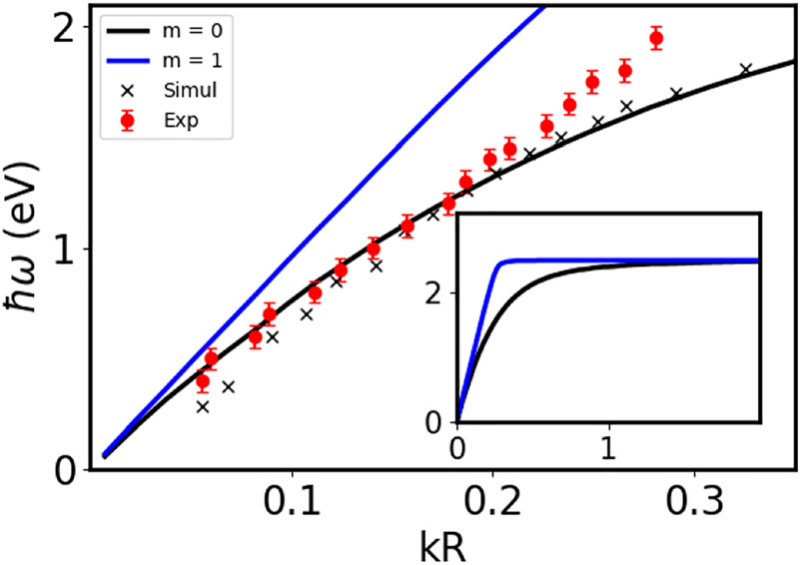
Dispersion relation of an infinite gold NW (*R* = 15 nm) for *m* = 0 (black curve) and *m* = 1 (blue curve) compared with the data obtained from EELS spectra for finite NW (*L* = 3 μm). × are for simulated EELS and 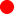 are for experimental EELS. The error bar indicates the 0.05 eV experimental spectral resolution. For the simulations, the dielectric function is taken from Ref. [52].

On Figure 3, the experimental and simulated dispersion relations associated with the FP modes of the 3 μm long NW (symbols) is compared with the dispersion relation of an infinite NW of the same radius (solid line), calculated using the retarded theory of the SPP on a cylinder [49] adapted for complex dielectric functions (see Supplementary Information). These simulations confirm that a finite size NW behaves like a FP cavity with no modification of the wavelength of the SPP. The good agreement between FP mode resonance energies and the SPP of the same wavenumber confirm that EELS experiments excite the totally symmetric *m* = 0 mode for an *e* ^im*ϕ*^ angular dependence of the induced field [43] (see also the induced field map in Supplementary Information). This totally symmetric mode can also be excited by light polarised along the NW axis and it is even the dominant mode in this case for small radius NWs [50]. The dipolar *m* = ±1 plasmon are the modes that are predominantly excited by light with a transverse polarisation. The observation of high intensity FP cavity modes requires that the SPP propagates on the NW with low losses and that the reflection probability at the extremity is close to one. The losses that occur at the tip of the NW, associated with the emission of light [51], and the damping of the SPP along the NW will both reduce the amplitude of the FP modes.

All the plasmon modes (for all *m*) rapidly converge to the planar surface plasmon energy at 2.45 eV for large *k* (see Figure 3 inset). This gives rise to the high energy peak (2.4 eV) in the EELS spectra. As these high *k* modes cannot be resolved, a continuous excitation probability is observed along the NW and at his extremity. The exact energy of this peak at 2.4 eV cannot be properly reproduced by the simulations because the dielectric response of gold is very dependent of the crystallinity of gold around this energy [52]. It is also worth mentioning that all the simulations in the present work are performed for self-supported nanosystems where the experimental EELS are obtained on an holey silicon oxide membrane (see Supplementary Information). The interaction with a dielectric substrate is known to induce a redshift of the plasmonic features [15, 24]. However, we have not observed a systematic disagreement of the simulated EELS response in comparison to the experiments. This is probably due to the small dielectric response of silicon oxide in the infra-red and visible (*n* ∼ 1.45). Other uncertainties (exact radius and length, shape of the extremities, numerical error due to the discretisation, …) are probably within the same order of magnitude, but still small.

**Figure:**
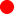


It is important to notice that, given the resolution of the data analysis and the very high aspect-ratio of the sample, the number of plasmonic modes seen in this Au NW is considerably higher when compared to the literature on the subject for these Au nanostructures [27]. We have also observed both in the experimental maps and in the simulated ones that, for higher energy modes, the EELS intensity is lower at the centre of the NW than at the edges as previously observed [29]. This is related to the lower propagation length of the SPP along the NW at excitation energy close to the volume plasmon resonance (see Supplementary Information), a phenomenon also observed for SPPs at planar interfaces [53].

### 2.2 Gold half-dumbbells and gold dumbbells

**Figure 4:**
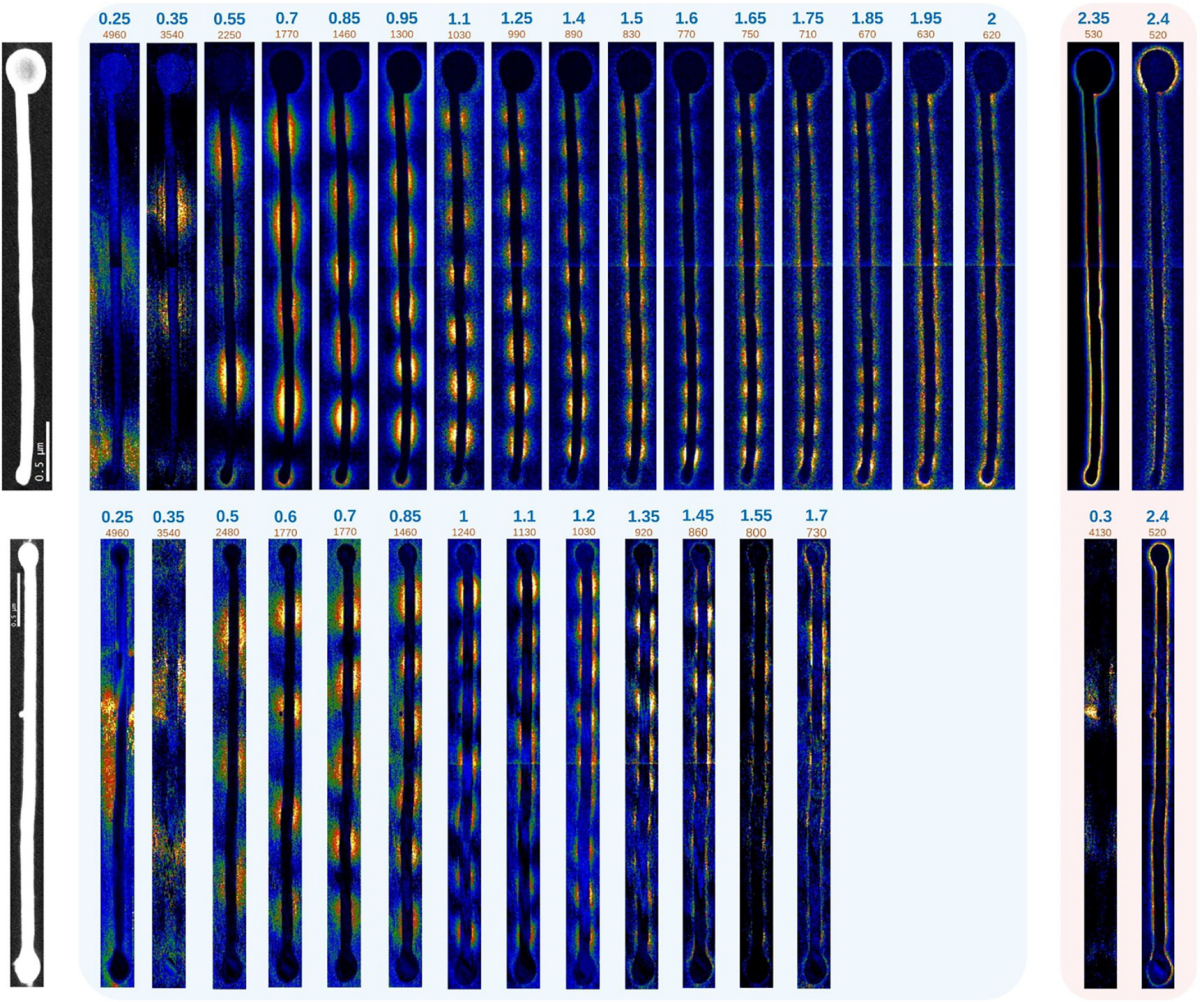
Spatial distribution of the plasmonic modes for the Au half-dumbbell (top) and the Au dumbbell (bottom). From left to right: STEM-HAADF image: NMF components corresponding to FP modes in ascending order of resonance energy (blue background), and NMF components corresponding to the surface modes of the Au NW and the Au NP at 2.35 and 2.4 eV for the half-dumbbell, and to the small attached nanoparticle and the whole system in the dumbbell, respectively.

The selected components from the NMF decompositions after background removal of the Au half-dumbbell (HDB) and the Au dumbbell (DB) spectrum-imaging STEM-EELS results are displayed in Figure 4. The Au half-dumbbell consists of a 3.2 ± 0.1 μm long nanowire of 110 ± 10 nm of diameter, attached to a nanoparticle of 340 ± 10 nm in diameter. Regarding the NW within the nanostructure, the NMF analysis allows the possibility to study both FP features, going from 0.25 to 2 eV; as well as other plasmonic resonances above 2 eV related to the Au nanowire and the Au nanoparticle, respectively. It is important to point out that it is possible to separate the mode located on the Au NW of the nanostructure (situated at 2.35 eV) from another mode located on the Au NP of the nanostructure (located at 2.4 eV). A similar localisation of the FP mode and of the LSPR of the tip has been performed on the simulated data (see Supplementary Information). This evidences the extreme sensitivity of our decomposition methodology.

The second Au nanostructure displayed in Figure 4 consists of a full dumbbell, resulting of joining a 3.58 ± 0.05 μm long NW with one nanoparticle in each end, each measuring 300 ± 10 nm and 210 ± 10 nm in diameter, respectively. The full dumbbell has a little 60 ± 10 nm diameter Au particle attached to it. Regarding the analysis of the measurements performed on this nanostructure, the FP modes can be discerned down to 0.25 eV. A feature at 0.3 eV has been found in our decomposition, associated with the small protrusion near the middle of the NW. It is related to an antenna effect of this protrusion on the lowest FP mode. This is another evidence of the possibility to differentiate both the energy of a EELS signature and its localisation based on our decomposition approach. This is of great importance for a detailed analysis of response of such objects.

Furthermore, the information from the surface modes indicates the presence of coupling between the NW and both NPs at the tips. The presence of coupling on the DB while there being a lack of coupling on the hDB can be explained by the difference in size of the nanoparticles in both structures.

**Figure 5:**
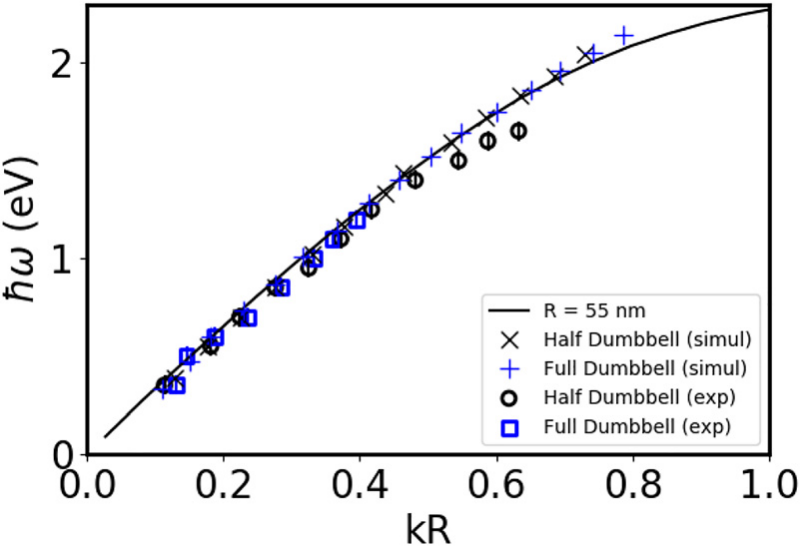
Dispersion of the *m* = 0 branch of infinite gold NW of *R* = 55 nm compared with the data extracted from the experimental (open squares and circles) and simulated (crosses) EELS spectra of finite size gold dumbbell and half-dumbbell. The error bars indicate the 0.05 eV experimental spectral resolution.

As for the NW without a nanoparticle at its extremities, the DDA simulations on the exact same geometry are in very good agreement with the NMF decomposition of the experimental EELS data. Indeed, Figure 5(a) displays the dispersion relations of both experimental and simulated plasmon excitation energies as a function of its wavenumber times *R* (*kR*) (symbols). The simulated EELS maps confirmed the agreement and are provided in the Supplementary Information.

**Figure 6:**
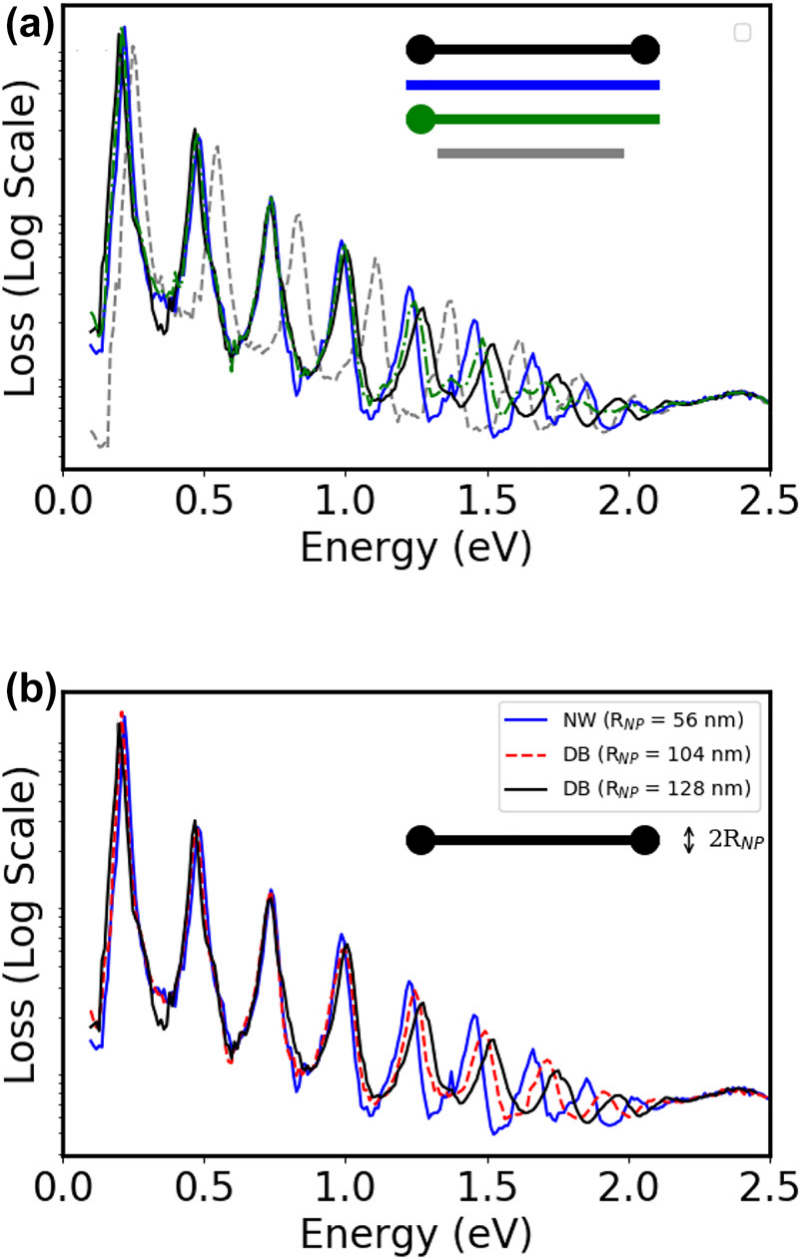
Simulated EEL spectra for different Au nanostructures: (a) Comparison of the simulated EELS loss spectra of NWs of total length *L* = 4140 nm for an aloof impact parameter at the mid-length: Dumbbell shape (Black), plain NW (Blue), half-dumbbell shape (dot-dashed green). The radius of the spheres at the extremity is 128 nm. The spectrum for a NW of length *L* = 3648 nm is displayed for comparison (grey dashed). (b) Simulated EELS spectra of plain NW and of dumbbell shape structures of total length *L* = 4140 nm with various radii of the spheres at the ends of the NW (*R*_NP_ = 104 nm and *R*_NP_ = 128 nm).

The solid lines on Figure 5 gives the analytical dispersion of the *m* = 0 of perfect infinite NW of radius of 55 nm (black curve). The dispersion relations deduced from the EELS data of finite DB and HDB follow it nicely. The SPPs can then be seen as intrinsic modes of the NW itself, regardless of the shape of the extremities. This brings us to the question of the role of the extremity on the FP modes. The comparison between the simulated EELS spectra of NW, DB and HDB of the same total length show very similar resonances for the low energy modes (Figure 6(a)). On the contrary, a comparison with a NW which length corresponds to the distance between the spherical extremities displays resonances at higher energies.

We further analyse the influence of the extremities on the FP modes of the DB on Figure 6(b), where the EELS spectra of NW (*R*_NP_ = 56 nm) is compared with the ones of DB for different diameters of the spherical ends (*R*_NP_ = 104 nm and *R*_NP_ = 128 nm). The influence of the modification of the extremity is clearly negligible for the low energy, low *k* (high wavelength) modes and starts to be noticeable when the wavelength of the FP is similar to the size of the perturbation.

**Figure 7:**
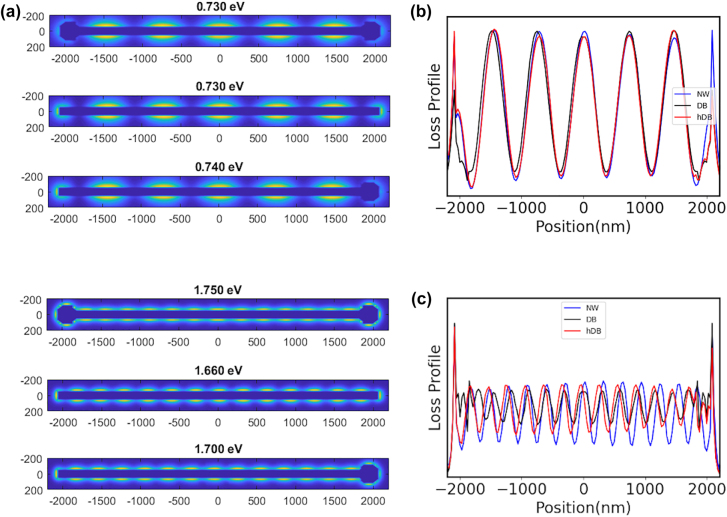
Simulated EELS maps and spectra profiles for the FP modes at two different energies: (a) Simulated EELS maps for the FP modes around 0.73 eV (first and second maps) and 1.7 eV (third and fourth maps), for the dumbbell with *R*_NP_ = 128 nm (first and third maps) and the plain NW (second and fourth maps). (b) and (c) EELS profiles.

This is also illustrated on Figure 7 where the simulated EELS map and the associated loss profile are detailed for two specific FP modes of the NW, the DB and the HDB systems. The FP mode at 0.73 eV (*n* = 5, *λ* = 1698 nm) is present for the three plasmonic systems with very similar EELS maps and loss profiles, including at the extremities of the NW. We can then deduce that both the propagation of the SPP and the reflection coefficient at the extremities are identical for the three cases. The change of the shape of the extremities, induced by the laser treatment, does not influence neither the energy of the plasmonic response of the NW, nor the field distribution (see Supplementary Information).

For the FP mode *n* = 13, the resonance occurs at *E* = 1.66 eV (*λ* = 607 nm) for the perfect NW, at *E* = 1.75 eV (*λ* = 560 nm) for the DB and at *E* = 1.70 eV (*λ* = 591 nm) for the HDB. First, the larger damping of the SPP (and lower propagation length) at this energy explains why the loss probability is lower for all the nanostructures than for the lower energy modes (see the loss profile). Second, in this case, the shape of the extremities plays an important role in the reflection of the SPP. The shorter wavelength (lower energy) of the *n* = 13 mode for the DB indicates that the reflection condition of the SPP are modified. A total reflection of the SPP on the edge of the spherical protrusions would lead to a wavelength of 518 nm for the DB (for a length between the spherical NPs of 3628 nm for a total length of 4140 nm and NPs radius of 128 nm). We are then in an intermediate configuration. This can be further analysed in terms of reflection coefficient. Besides the reduced loss oscillation magnitude, a change in the imaginary part of the reflection coefficient attributed to the shape and size of the nanowire ends can explain the dephasing and shortening of loss oscillations when close to the tips, in comparison to the NW case. It is reminded that the oscillation phase is imposed by the excitation of the SPP by the electron beam. The role of this reflection is further evidence is the profile of the HDB. The position of the maximum loss probabilities follows the pattern of the perfect NW on the perfect extremity where it follows the pattern of the DB on the side of the spherical protrusion. This highlights also to role of the shape and of the size of the nanoparticles at the tip of the NW on the exact FP mode energies for small wavelength. The correct description if this shape and size is then important to predict the exact energy of these modes. This is also illustrated by the dependence of the loss spectra with the size of the spherical NP (Figure 6b). This gradual shift of the FP modes energies can be associated with a continuous modification of the reflection coefficient with the size of the extremities. This explain why the match between the simulations and the experimental data are less good for the HDB, which displays a less spherical extremity and a small modification of the other end (Figure 5).

We have then observed and rationalised that the modification of high aspect ratio NW by laser irradiation allows to tune specifically large *k*, small *λ* modes, while the low energy modes are not perturbed.

## 3 Conclusions

Laser-induced tuning of the plasmonic response in Au high aspect ratio nanostructures has been reported by both its deep study via low-loss STEM-EELS measurements and theoretical modelling by use of discrete dipole approximation calculations. We evidence that the modification of the extremities of a high aspect ratio metallic NW allow the control the resonance energy for small wavelength plasmon modes where longer wavelength modes stay almost unperturbed. These works establish an initial road-map for a detailed tuning by laser irradiation of the energy of small wavelength Fabry–Pérot modes in these nanostructures. On the other hand, an robustness of the dielectric response of the NW against the modification of the extremities reinforce their potential interest as nanophotonic waveguides and low energy resonators. These findings demonstrate that high aspect ratio nanostructures are very interesting candidates for future nanophotonic applications.

## Supplementary Material

Supplementary Material Details
