# Supplementary material for: Toward laser-induced tuning of plasmonic response in high aspect ratio gold nanostructures
Source: Nanophotonics. 2022 Aug 8;11(16):3719–28. doi: 10.1515/nanoph-2022-0193 (PMC11501715; doi:10.1515/nanoph-2022-0193)
Supplement: Supplementary file 1 — Supplementary Material Details [file j_nanoph-2022-0193_suppl.pdf]

Research Article

Mario Pelaez-Fernandez, Bruno Majerus, Daniel Funes-Hernando, Romain Dufour, Jean-Luc Duvail*, Luc Henrard*, and Raul Arenal*

Supporting Information: Toward Laser-Induced Tuning of Plasmonic Response in High Aspect Ratio Gold Nanostructures

https://doi.org/10.1515/sample-YYYY-XXXX
Received Month DD, YYYY; revised Month DD, YYYY; accepted Month DD, YYYY

1 Materials and methods

1.1 Au NW fabrication

The fabrication of the gold nanowires (Au NW) was performed using a templated electrochemical synthesis [1] as presented in previous works [2]. Anodic aluminium oxide porous membranes (AAO) were used as a template for this synthesis. The size of the pores in the membrane dictated the diameter of the NW. Electroplating of gold within nanopores was made by cyclic voltammetry (0.00–0.75 V vs. saturated calomel electrode SCE, scan rate: $50mVs^{-1}$) applied to an aqueous electrolyte of chloroauric acid (0.03 M), K_2HPO_4 (0.1M) and KCl (0.1 M). The length of the nanowires was controlled by the number of cycles. Ultrapure water (resistivity $\rangle$ 18.2 MΩ cm) was used in all the steps of the process. The AAO membrane removal was achieved by immersion in a 4.9 M H_3PO_4 solution for 48 hours. The resulting array was rinsed with ultrapure water several times. After a sonication step to disperse the Au NW in water, the resulting Au NW dispersion was drop cast into a membrane of silicon oxide with holes supported on a copper TEM grid.

1.2 Au dumbbell fabrication

The dumbbell structures were obtained by laser heating on the tips of either one or two of the ends of the gold nanowire once it was deposited on the substrate [3]. This laser irradiation provoked the gold melting at the ends of the NW and the formation of gold nanoparticles attached to the NW, hence producing a high aspect-ratio dumbbell or half-dumbbell, respectively [4]. This has been achieved by using a 488 nm laser source with a power of 15 mW applied during 3 seconds through a x100 long focal objective with a numerical aperture of 0.8.

Mario Pelaez-Fernandez, Raul Arenal, Instituto de Nanociencia y Materiales de Aragon (INMA), CSIC-U. de Zaragoza, Calle Pedro Cerbuna 12, 50009 Zaragoza, Spain
Mario Pelaez-Fernandez, Raul Arenal, Laboratorio de Microscopias Avanzadas, Universidad de Zaragoza, Calle Mariano Esquillor, 50018 Zaragoza, Spain, e-mail: mariopf@unizar.es, arenal@unizar.es
Bruno Majerus, Romain Dufour, Luc Henrard, Laboratoire de Physique du Solide, NISM, University of Namur. 61, rue de Bruxelles 5000 Namur, Belgium, e-mail: bruno.majerus@unamur.be, romain.dufour@uclouvain.be, luc.henrard@unamur.be
Daniel Funes-Hernando, Jean-Luc Duvail, Institut des Matériaux de Nantes Jean Rouxel, CNRS- Université de Nantes, France, e-mail: dfunesher@gmail.com, jean-luc.duvail@cnrs-imn.fr
***Corresponding author: Raul Arenal,** ARAID Foundation, 50018 Zaragoza, Spain, e-mail: arenal@unizar.es

1.3 TEM - EELS measurements and data analysis

STEM images and spatially resolved EELS spectrum-images (SPIMs) [5] were taken using a FEI Titan Low-base microscope working at 80 keV, equipped with an ultrabright X-FEG electron source, a Cs probe corrector, a Gatan Tridiem 866 ERS energy filter and a monochromator, offering an energy resolution down to 160 meV. The convergence and collection angle were 25 and 35 mrad, respectively. As for the SPIM-EELS acquisitions, the acquisition time has been set at 0.01 s/pixel (10 EEL spectra of 10 ms/each have been recorded per pixel). EELS-SPIMs were acquired using a subnanometer electron probe that was rastered over the region of interest with a step size of 7.9 – 8.1 nm. After collecting the data, each EEL spectra was aligned using the zero-loss peak (ZLP). The analysis of the EELS results has been developed using a custom ZLP removal algorithm as well as a multivariate decomposition technique, non-negative matrix factorization (NMF) [6, 7], to obtain the plasmonic response of these nanostructures, which presented a difficult analysis given the spectroscopic properties of this kind of structures [8–10]. Both analyses are explained in depth in the following section.

1.4 EELS simulations by discrete dipoles approximation

Numerical simulation of EELS spectra and optical properties have been performed within the DDA method, as described in ref [11] with the code DDEELS. In brief, the metallic particles is described by a set of dipole with an associated polarisability obtained from the bulk dielectric function of gold [12] by means of the Clausius-Mossotti formula. In the present study, the number of dipoles is taken between 38000 for the smaller nanowire and 110000 for the longer dumbbell, with a discretisation ranging from 4 to 8 nm. The set of coupled equations have been solved iteratively with the use of the Fast Fourier Transform algorithm [13]. Spectra and EELS loss intensity maps are computed as well as maps of the induced electro-magnetic field and of the induced charge densities.

1.5 EELS data analysis

Due to the high aspect ratio of these samples, there is a higher concentration of the various plasmonic features, which are closer in energy to one another than their low aspect-ratio counterparts[8–10]. This, on the one hand, makes it impossible to find a region with no plasmonic activity to extract the background from. On the other hand, this also makes it challenging to discern the different eigenvalues of the plasmonic resonances due to overlapping. This overlapping occurs both among Fabry-Perot (FP) modes and between FP modes and the main fully symmetric ($m = 0$) modes of the sample, as it can be seen in Figures 1 and 2. The impossibility to find a spectral window to extract the background from leaves out the option for a power law background removal, often used for this kind of studies. For this reason, a custom background removal routine has been set in place for the analysis of these particular objects.

This routine has consisted on creating a spectral reference by integrating an average of 200 spectra taken from the edges of each spectrum-image (where a null or very low plasmonic activity is assumed) and using this spectral reference as a component to subtract from each spectrum in the SPIM, with the condition that the resulting spectrum needs to be positive for each energy value. Both the different spectra in the SPIM and the spectral reference have been re-binned by a factor of 2, and the positivity condition has only been upheld for the energy window between 0.2 and 16.2 eV. Both of these measures have been taken in order to diminish the effect of the spectral noise on the background removal. An integrated spectrum after the removal of the ZLP is shown in the main manuscript.

Once the background has been removed, the overlapping between the different plasmonic features complicates the differentiation of the different plasmonic modes for their subsequent mapping. In order to gain a better understanding on the behavior of these nanostructures, NMF decomposition has been used to discern between the different plasmonic modes, as it has been seen in the literature [14]. Due to the amount

of signal-to-noise ratio (SNR), and its variation with energy loss, this decomposition could not be performed for the whole SPIM at once. Instead, it has been performed on 1eV-wide spectral windows with a step of 0.5 eV in order to increase the accuracy of the decomposition. Relevant modes have been then selected from each 16-component decomposition. An example of the different spectra and their NMF decompositions at two given positions in the SPIM can be seen in Figure 1.

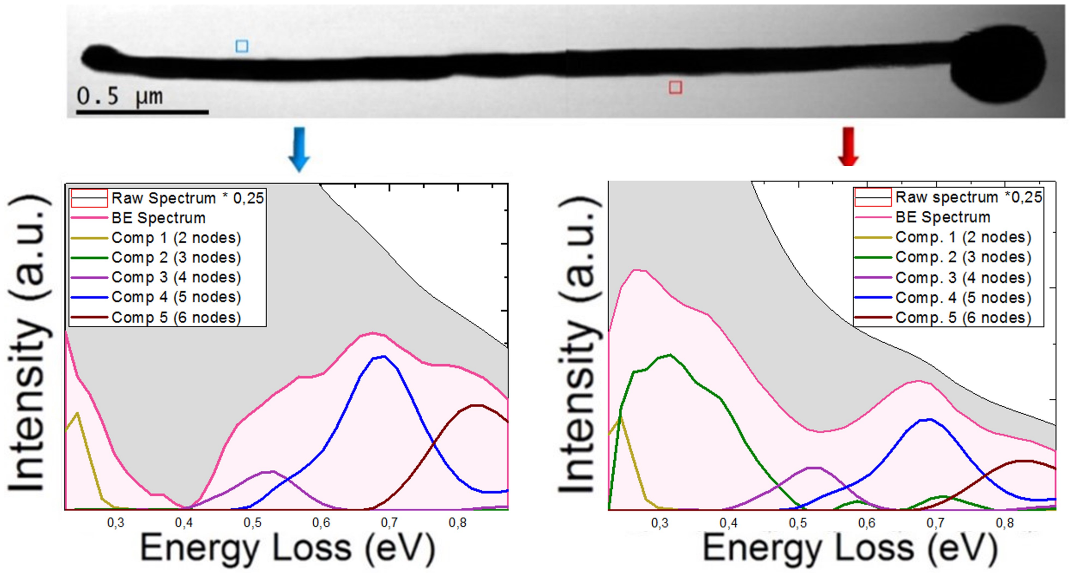

Fig. 1: Low-loss EELS NMF spectral decomposition on two different regions of a given EELS SPIM. The comparative between these two points in the SPIM shows the great deal of overlapping taking place between the different plasmonic components in the spectra.

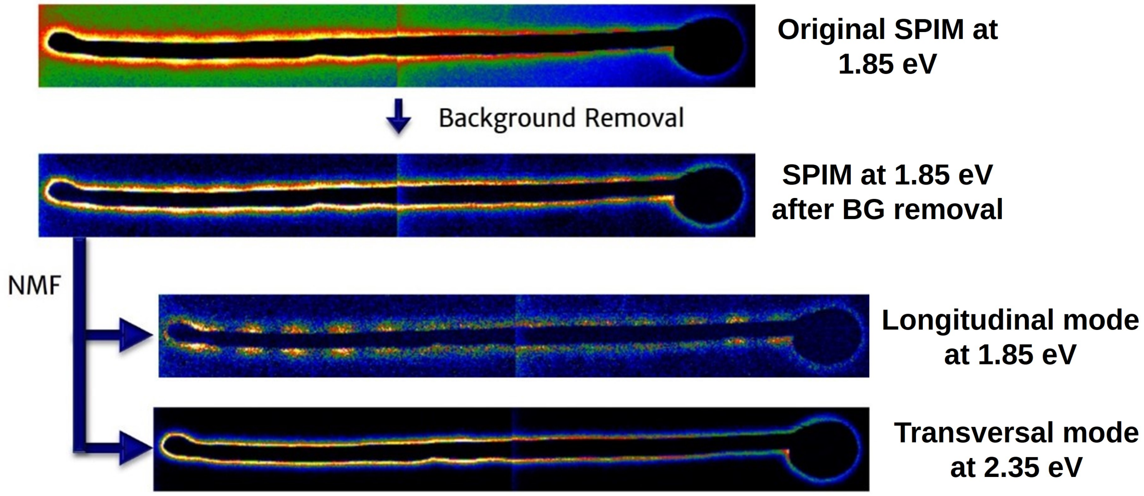

Fig. 2: Schematic for the TEM-EELS analysis process at a given energy. From top to bottom: Energy map at 1.85 eV from the original SPIM, energy map after background removal and the FP and transversal modes that appear overlapped at this energy.

1.6 Data analysis of the simulated EELS

There is one particular challenge when modelling the plasmonic response of gold nanostructures. The dielectric response of gold shows a sharp change around 2.5 eV that translates into step increases of the real and imaginary parts of the dielectric function. The exact response of the nanostructures depend on the crystallinity (and thus on the growth process). Consequently, the assignment of the observed peaks in the EELS spectra in this energy region is particularly challenging for both experiments and simulations because of the overlapping of the surface plasmon modes. However, as seen in Figure S3, the shift in energy between the m=0 mode at the NW and at the NP is clear even for the raw EEL spectra, so those two modes should be separated in the DDA analyses as well.

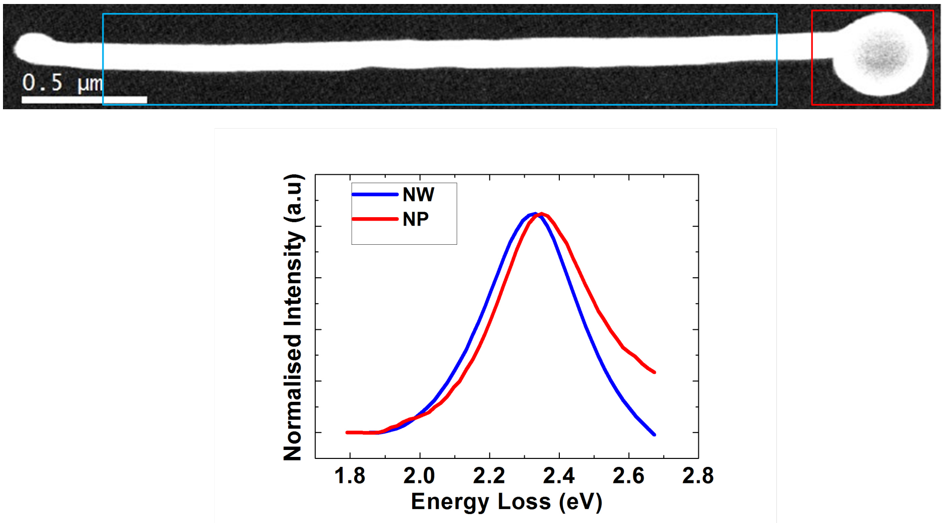

Fig. 3: Integrated EEL spectra around the NW and the NP for the spectral range between 2 and 2.8 eV. Results show a clear shift between the NW mode and the NP mode.

For a better analysis of the EELS map simulated by DDA, several intensity maps were calculated at different energies (2.2, 2.25, 2.3, 2.35, 2.4 and 2.45 eV) and then merged onto a data cube that was treated following the same NMF decomposition as their experimental counterpart. The results for a 2-component decomposition of these modelled results can be seen in Figure S3.

This decomposition shows that there is also a presence of a NW mode and a NP mode in the model, which is coherent with our experimental results.

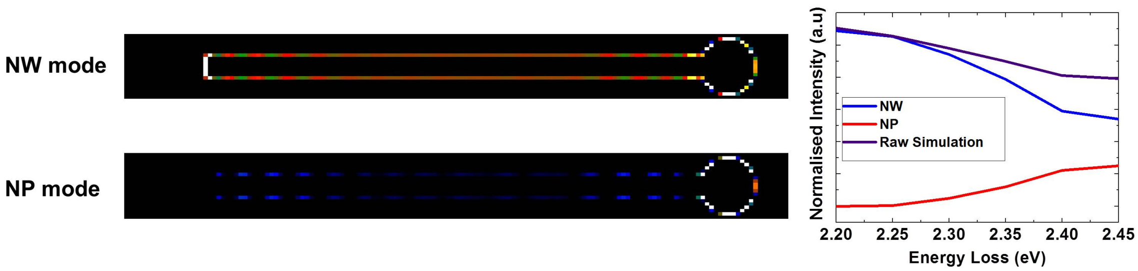

Fig. 4: 2-component NMF decomposition of the data cube conformed by merging different simulated intensity EELS maps at different energies.

This decomposition shows that there is a presence of a component located in the Au NW mode and another one located at the Au NP in the model, which is coherent with our experimental results.

2 Dispersion of plasmon polariton of infinite nanowires

For an infinite cylinder, the azimuthal dependence of the fields is $e^{im\phi}$ and the dependence along the axis is e^{ikz}. Following the work of Ashley and Emerson [15], the dispersion relation of the surface polariton of an infinite cylinder of radius a described by a real dielectric function $\varepsilon(\omega)$ in a medium $\varepsilon'(\omega)$ can be obtained from the following equation:

$$v^2 v'^2 \left(v'\varepsilon\alpha_m - v\varepsilon'\beta_m\right)\left(v'\alpha_m - v\beta_m\right) - \frac{m^2}{a^2}\left(\varepsilon' - \varepsilon\right)^2 k = 0 \tag{1}$$

with k the wavevector along the NW axis and m the azimuthal number and

$$\begin{aligned} v &= \left(k^2 - \varepsilon\omega^2/c^2\right)^{\frac{1}{2}} \\ v' &= \left(k^2 - \varepsilon'\omega^2/c^2\right)^{\frac{1}{2}} \\ \alpha_n &= \frac{\mathrm{d}}{\mathrm{d}(va)} \ln I_n(va), \\ \beta_n &= \frac{\mathrm{d}}{\mathrm{d}\left(v'a\right)} \ln K_n\left(v'a\right) \end{aligned} \tag{2}$$

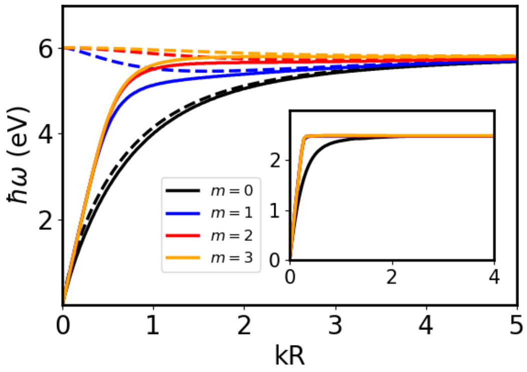

Fig. 5: Dispersion of plasmon polariton branches m= 0,1,2,3 of an infinite gold nanowire of radius $R = 15$ nm as a function of kR (solid lines) compared with the non-retarded calculations (dashed lines). A Drude model with $\omega_P = 8.5$ eV is used. Inset : Dispersion of plasmon polariton for tabulated dielectric data [12].

The function I_n and K_n are the modified Bessel functions. For a complex dielectric function, we have taken the maximum of the imaginary part of the left member of the equation 1. The figure 5 compare the dispersion of the infinite NW for a Drude mode (main panel) and the actual tabulated dielectric function of Gold (inset) for R = 15 nm. The correspondence is very good up to 1.2 eV and the dispersion curves diverge for higher energy, due to the peculiar behaviour of the dielectric function of noble metal related to the d orbital transition.

On the same figure, we show the dispersion obtained analytically in the non-retarded approximation for a the same Drude model (dashed line) that writes for $\varepsilon'(\omega) = 1$ (Ref [16])

$$\omega_m(k)^2 = -\omega_{\mathrm{p}}^2 kRI_m(ka)K_m'(ka) \tag{3}$$

For such small radii ($R = 15$nm) non retarded dispersion curve for $m = 0$ gives a very good approximation of the actual retarded dispersion relation. An illustration of the role of the NW radius of the dispersion of NW as a function of kR is presented on Figure 6 where the $m = 0$ mode of non-retarded (dashed line) and retarded (solid lines) dispersion for $R = 15$, 30, 50 and 100 nm are presented. The non-retarded curve is universal and only depends on kR (Eq. 3).

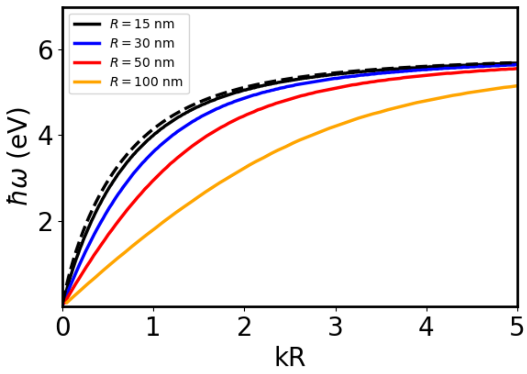

Fig. 6: Dispersion of plasmon polariton branches m= 0 of an infinite gold nanowire of various radii as a function of kR (solid curves) compared with the non-retarded calculations (dashed lines). A Drude model with $\omega_P = 8.5$ eV is used.

3 Link between infinite NW and finite nanosystems

The wave vector associated with each modes could been deduced from

$$k_n = n' \frac{\pi}{L} \tag{4}$$

with n' the number of nodes and L the total length. However, as explain in the main text, the high energy modes are modified by the presence of a nanoparticle at the extremity of the NW. For each nanostructure, we then take the reference length as the length between the first and the last anti-node, L_{AN} and

$$k_n = (n-1) \frac{\pi}{L_{AN}} \tag{5}$$

With $n' = n + 1$. An example for one of the modes in the half-dumbbell is shown in Figure 7 for reference.

For perfect NW and low energy modes for DB and HDB, both ways to determine k are equivalent.

However, for experimental data analysis, the slight curvature of the sample make difficult this approach. A custom script has been created to compare the HAADF-STEM intensity of the SPIM, find the edge of the NW and create a profile with the immediate pixels to said edge. From then on, the process to measure the value of λ has been the same as the other samples. The model results had a higher SNR; therefore, a more analytical approach could be used. A profile was extracted from each modelled map and fitted using a Gaussian multi-peak fit.

4 Comments on the $m = 0$ modes

In a non-retarded approach, the EELS spectra of NW take the following general form [17].

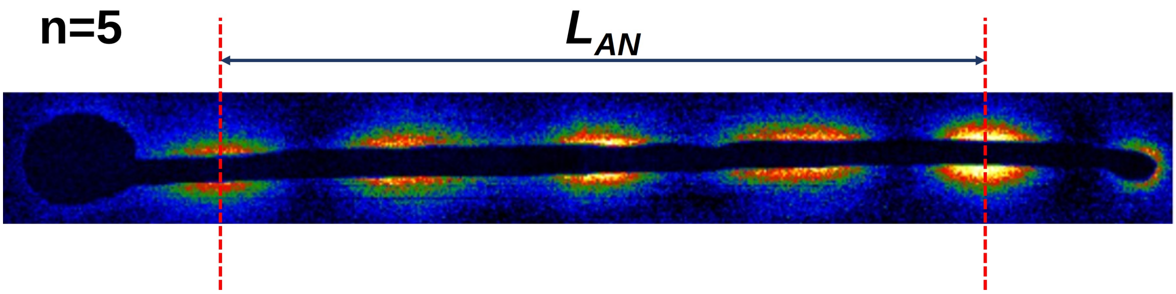

Fig. 7: Schematic representation for the parameters gathered from the half-dumbbell plasmonic mode at 0.85 eV, notably n=5 (the number of antinodes taken for the estimation of λ) and L_{AN} (the distance separating the center of the two outermost antinodes). In this example, $n' = 6$

$$I(\omega, b) = \frac{q^2}{2\pi^3 \epsilon_0 \hbar^2} \times \sum_{m=-\infty}^{+\infty} \int_0^{+\infty} dk \, \mathrm{Im}\left[\alpha_{m,k}(\omega)\right] C^2_{m,k}(\omega, b) \tag{6}$$

with b the impact parameter and q the electron charge. $\alpha_{m,k}(\omega)$ is the response function (polarisability) intrinsic to the NW. $C_{m,k}(\omega, b)$ is directly related to the electron beam trajectory and is independent of the NW. It acts as a filter for the mode excitations, i.e. it modulates the excitation of a given (m, k) mode as a function of the experimental conditions. Similar expressions for the loss of a NW are also given in Refs. [16, 18] but, to the best of our knowledge, are not available for retarded approach. Numerical simulations on a finite NW presented in this study have been performed in a retarded approach.

In the present experimental results and in the simulations on finite NW, only the $m = 0$ mode is excited for each k as confirmed by the very good agreement between the dispersion curve of infinite NW and the FP standing waves of finite NW but also by the induced charge density (see Figure 8). This map is similar to the one in Ref. [19].

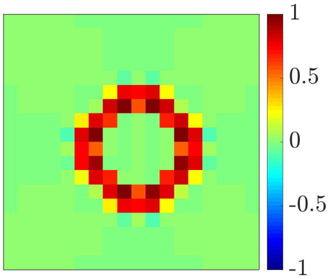

Fig. 8: Simulated induced charge density (section of the NW) of the 0.6 eV mode of the EELS spectra of the gold NW. See Fig 2 in the main text for the corresponding EELS map.

From the retarded dispersion relations (i.e. Fig.6), we see that the $m > 0$ modes appears at the same energy range as the $m = 0$ modes (but for different k) and could then be potentially observed by EELS. There is also no selection rules or symmetry consideration that preclude their excitations and, moreover, the $m = \pm 1$ are excited by light [20] and observed by cathodoluminescence [21]. We postulate that, in our case, the $m = 0$ is very dominantly excited for all impact parameters because of the very dominant intrinsic response of this mode. This is corroborate by the fact that the $C_{m,k}$ factor is not much larger for the $m = 0$

mode. It could be related to the small radius of the NW in the same way the $m = 0$ mode is predicted to be dominant in optical experiments in this case [20].

5 Comparisons between experimental and simulated EELS map for dumbbell and half-dumbbell nanosystems

Figure 9 and Figure 10 show the modelled energy loss maps for the different plasmonic modes in the Au half-dumbbell and the Au dumbbell, respectively. There is a very good agreement between the experimental and the modelled maps, even in the case of the Au dumbbell, which had the lowest SNR out of all the analysed samples.

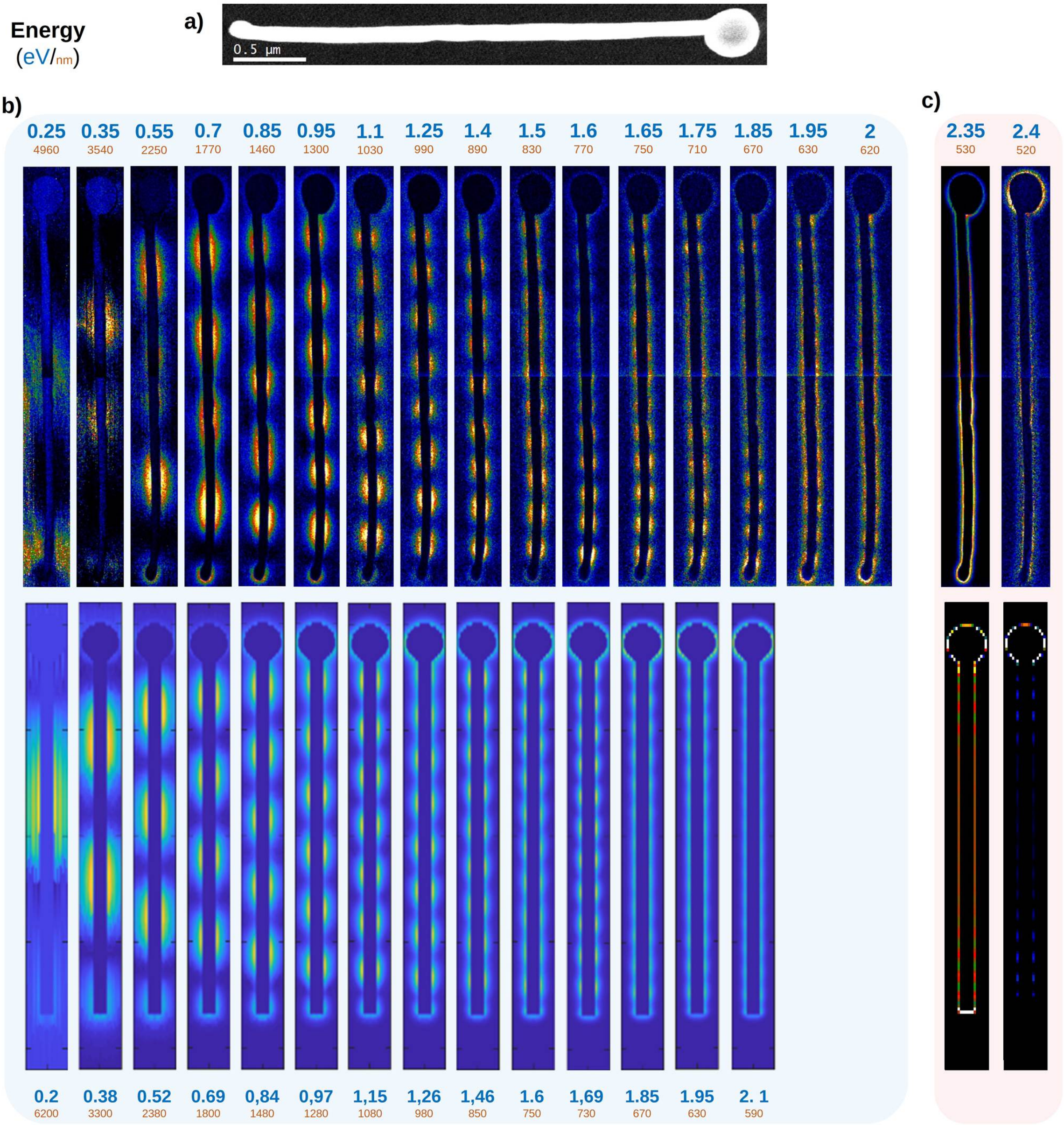

Fig. 9: Plasmonic analysis of the half-dumbbell system obtained by background extraction and NMF decomposition. (a) STEM-HAADF micrograph, (b) Top: NMF components corresponding to FP modes in ascending order of resonance energy. Bottom: DDA simulations corresponding to FP modes with the same number of nodes as their experimental counterparts. (c) Top: NMF components corresponding to the transversal mode of the Au NW and the Au NP of the half-dumbbell, respectively. Bottom: DDA simulations.

6 Absence of wavelength contraction

A wavelength contraction phenomena has been previously reported [9, 14, 22] but not mentioned in other publication [19]. We do not observe such behavior in our experimental results or in our simulations. Further details are given on Fig 11 where the distance from one anti-node to the other is given for the two modes analyzed on Fig. 7 of the main text. Except for the last anti-node that is influenced by the extremity, the wavelength of the FP mode is constant all over the NW.

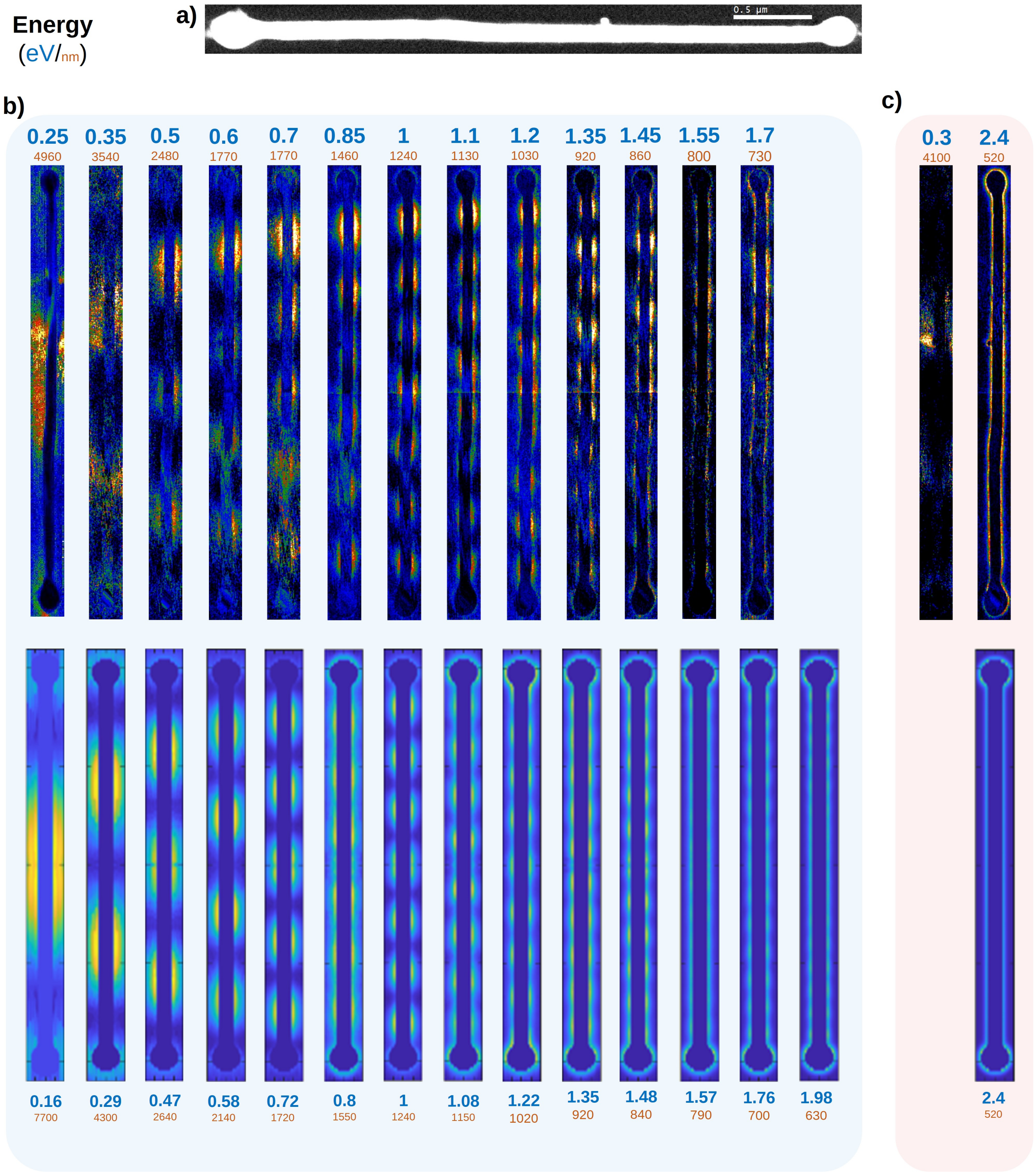

Fig. 10: Plasmonic analysis of the dumbbell system obtained by background extraction and NMF decomposition. (a) STEM-HAADF micrograph, (b) Top: NMF components corresponding to FP modes in ascending order of resonance energy. Bottom: DDA simulations corresponding to FP modes with the same number of nodes as their experimental counterparts. (c) Top: NMF components corresponding to the transversal mode of the dumbbell and the Au NP attached to the system, respectively. Bottom: DDA simulations.

7 Induced field and propagation length

For a better analysis of the propagation length of the FP plasmon mode on a finite size NW, the Fig 12 present the induced electric field by a electron beam impinging at the bottom end of the NW. The low energy modes (long wavelength) propagate with almost no loss along the tube. The induced electric

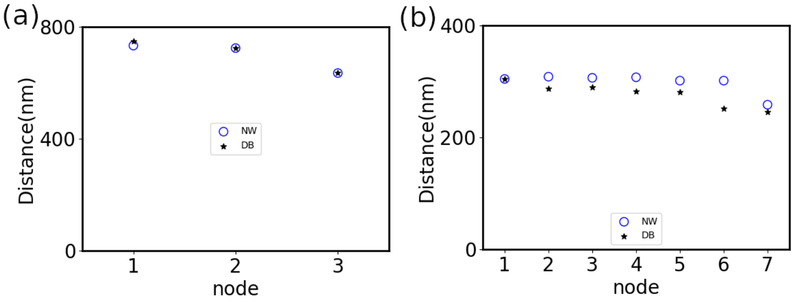

Fig. 11: Comparison of distance from one anti-node to the other is given for the two modes analyzed on Fig. 7 of the main text.

field of the high energy modes (short wavelength) decrease with the distance to the electron. This energy dependence of the propagation length of SPP is similarly observed in SPP of planar interface [23].

This behavior is at the origin of the lower loss intensity for the FP mode of high energy at the center of the NW (see main text). Indeed, the amplitude of the FP modes are related to the amplitude of the surface plasmon that propagates to the end of the tubes, reflects on it and propagates back to the excitation point.

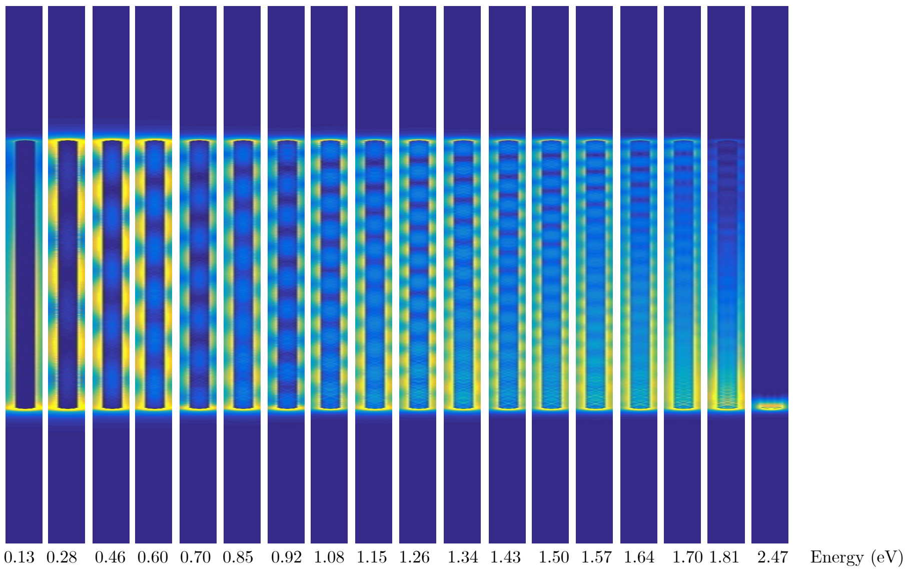

Fig. 12: Norm of the induced electric field at different loss energies for an electron beam impinging at the bottom ends of the NW.

The induced field by an electron external trajectory at half length of the NW is displayed on Fig. 13. For the low energy mode (Fig. 13 a), the influence of the spheres at the extremities is small and the profile of the electric field are similar. For the high energy mode (Fig. 13b), the decrease of the induced field from the extremities to the center could be directly related to the lower propagation length (larger damping) already observed in Fig 12. The mode is exited by the electron beam passing aloof the center of the NW, the SPP have then to travel twice the distance from the center to the extremities to establish a FP mode. For the DB and HDB system, the reflection probability at the extremities is smaller (related to the higher light emission) and the FP mode has a lower amplitude. For the HDB, it is striking that the induced field maxima on the left part occur at the same position as for the perfect NW where it is similar to the DB case on for the right part. This evidence the role of the reflection of the SPP on the extremities of the system.

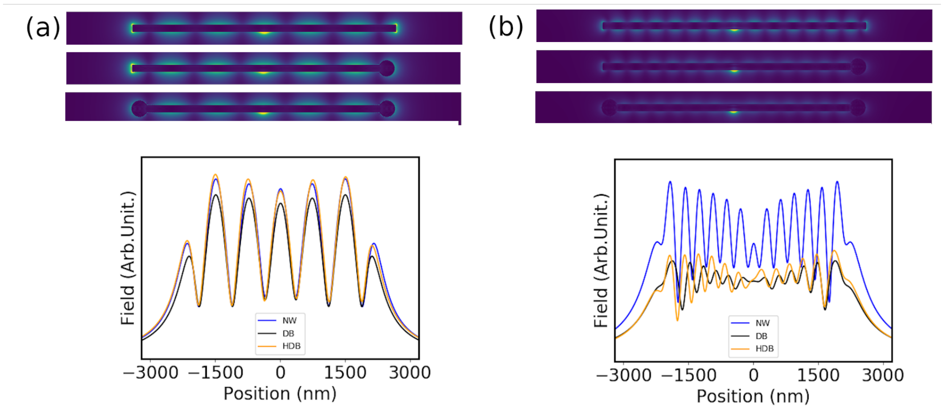

Fig. 13: Norm of the induced electric field resulting from the excitation of the FP mode around 0.74 eV (a) and around 1.70 eV (b) by an external electron beam passing at half length of the NW. Top : Map in the central plane, Bottom : line profile.

References

[1] Tuncay Ozel, Gilles R. Bourret, and Chad A. Mirkin. Coaxial lithography. *Nature Nanotechnology*, 10(4):319–324, 2015.

[2] D. Funes-Hernando, M. Pelaez-Fernandez, D. Winterauer, J. Y. Mevellec, R. Arenal, T. Batten, B. Humbert, and J. L. Duvail. Coaxial nanowires as plasmon-mediated remote nanosensors. *Nanoscale*, 10(14):6437–6444, 2018.

[3] Sergey V. Makarov, Anastasia S. Zalogina, Mohammad Tajik, Dmitry A. Zuev, Mikhail V. Rybin, Aleksandr A. Kuchmizhak, Saulius Juodkazis, and Yuri Kivshar. Light-Induced Tuning and Reconfiguration of Nanophotonic Structures. *Laser and Photonics Reviews*, 11(5):1–25, 2017.

[4] D. Funes-Hernando. *From hybrid structures to original plasmonic and optical properties*. PhD thesis, Université de Nantes, 2018.

[5] C. Jeanguillaume and C. Colliex. Spectrum-image: The next step in EELS digital acquisition and processing. *Ultramicroscopy*, 28(1-4):252–257, apr 1989.

[6] Yu-Xiong Wang and Yu-Jin Zhang. Nonnegative Matrix Factorization: A Comprehensive Review. *IEEE Transactions on Knowledge and Data Engineering*, 25(6):1336–1353, jun 2013.

[7] Alberto Eljarrat, Sònia Estradé, and Francesca Peiró. Low-loss EELS methods. 209:49–77, 2019.

[8] D. Rossouw, M. Couillard, J. Vickery, E. Kumacheva, and G. A. Botton. Multipolar Plasmonic Resonances in Silver Nanowire Antennas Imaged with a Subnanometer Electron Probe. *Nano Letters*, 11(4):1499–1504, apr 2011.

[9] David Rossouw and Gianluigi A. Botton. Plasmonic response of bent silver nanowires for nanophotonic subwavelength waveguiding. *Physical Review Letters*, 110:1–5, 2013.

[10] Sean M. Collins, Olivia Nicoletti, David Rossouw, Tomas Ostasevicius, and Paul A. Midgley. Excitation dependent Fano-like interference effects in plasmonic silver nanorods. *Physical Review B*, 90(15):155419, oct 2014.

[11] Nicolas Geuquet and Luc Henrard. EELS and optical response of a noble metal nanoparticle in the frame of a discrete dipole approximation. *Ultramicroscopy*, 110(8):1075–1080, jul 2010.

[12] Robert L. Olmon, Brian Slovick, Timothy W. Johnson, David Shelton, Sang Hyun Oh, Glenn D. Boreman, and Markus B. Raschke. Optical dielectric function of gold. *Physical Review B - Condensed Matter and Materials Physics*, 86(23):1–9, 2012.

[13] J. J. Goodman, B. T. Draine, and P. J. Flatau. Application of fast-fourier-transform techniques to the discrete-dipole approximation. *Opt. Lett.*, 16(15):1198–1200, Aug 1991.

[14] Olivia Nicoletti, Martijn Wubs, N. Asger Mortensen, Wilfried Sigle, Peter A. van Aken, and Paul A. Midgley. Surface plasmon modes of a single silver nanorod: an electron energy loss study. *Optics Express*, 19(16):15371, 2011.

[15] J.C. Ashley and L.C. Emerson. Dispersion relations for non-radiative surface plasmons on cylinders. *Surface Science*, 41(2):615–618, feb 1974.

[16] G. F. Bertsch, H. Esbensen, and B. W. Reed. Electron energy-loss spectrum of nanowires. *Phys. Rev. B*, 58:14031–14035, Nov 1998.

[17] D. Taverna, M. Kociak, V. Charbois, and L. Henrard. Electron energy-loss spectrum of an electron passing near a locally anisotropic nanotube. *Phys. Rev. B*, 66:235419, Dec 2002.

[18] A. Rivacoba, P. Apell, and N. Zabala. Energy loss probability of stem electrons in cylindrical surfaces. *Nuclear Instruments and Methods in Physics Research Section B: Beam Interactions with Materials and Atoms*, 96(3):465–469, 1995. The Interaction of Swift Particles and Electromagnetic Fields with Matter.

[19] Vahagn Mkhitaryan, Katia March, Eric Nestor Tseng, Xiaoyan Li, Leonardo Scarabelli, Luis M. Liz-Marzán, Shih Yun Chen, Luiz H.G. Tizei, Odile Stéphan, Jenn Ming Song, Mathieu Kociak, F. Javier García De Abajo, and Alexandre Gloter. Can Copper Nanostructures Sustain High-Quality Plasmons? *Nano Letters*, 21(6):2444–2452, 2021.

[20] Shunping Zhang, Hong Wei, Kui Bao, Ulf Håkanson, Naomi J. Halas, Peter Nordlander, and Hongxing Xu. Chiral surface plasmon polaritons on metallic nanowires. *Phys. Rev. Lett.*, 107:096801, Aug 2011.

[21] R Gómez-Medina, N Yamamoto, M Nakano, and F J García de Abajo. Mapping plasmons in nanoantennas via cathodoluminescence. *New Journal of Physics*, 10(10):105009, oct 2008.

[22] Jérôme Martin, Mathieu Kociak, Zackaria Mahfoud, Julien Proust, Davy Gérard, and Jérôme Plain. High-resolution imaging and spectroscopy of multipolar plasmonic resonances in aluminum nanoantennas. *Nano Letters*, 14, 10 2014.

[23] Stephan A. Maier. *Plasmonics. Fundementals and Applications*. Springer, Bath, 2007.
